# Systemic inflammation prevalence in patients with atherosclerotic cardiovascular disease and chronic kidney disease: a population-based study using a nationwide primary care database in Spain

**DOI:** 10.3389/fcvm.2025.1538466

**Published:** 2025-03-04

**Authors:** Giancarlo Pesce, Gaelle Gusto, Pierre Johansen, Artak Khachatryan, Bernabe Lopez-Ledesma, Jelena Vukmirica, Aleix Cases

**Affiliations:** ^1^ Real-World Evidence & Modeling Solutions, Certara, Milan, Italy; ^2^ Real-World Evidence & Modeling Solutions, Certara, Paris, France; ^3^ External Affairs, Novo Nordisk A/S, Copenhagen, Denmark; ^4^ Real-World Evidence & Modeling Solutions, Certara, London, United Kingdom; ^5^ Cardiovascular Disease Department, Novo Nordisk Pharma S.A., Madrid, Spain; ^6^ Medical Affairs, Novo Nordisk A/S, Copenhagen, Denmark; ^7^ Servicio de Nefrologia, Hospital Clínic, Barcelona, Spain

**Keywords:** atherosclerotic cardiovascular disease, chronic kidney disease, systemic inflammation, C-reactive protein, undertreatment

## Abstract

**Introduction:**

Systemic inflammation is recognised as a critical driver of atherosclerotic cardiovascular disease (ASCVD), especially in patients with comorbid chronic kidney disease (CKD). This study aims to assess the prevalence of systemic inflammation in the ASCVD population in Spain.

**Methods:**

Outpatient electronic medical records from The Health Improvement Network (THIN®) database were used to identify patients with ASCVD and a C-reactive protein (CRP) measurement ≥1 between January 2014 and July 2023 in Spain. The proportion of patients with systemic inflammation (defined as CRP ≥ 2 mg/L) was estimated at the first CRP measurement (index date) and at the end of the study. The patients' characteristics, comorbidities, and drug dispensation in the prior 12 months were reported by systemic inflammation status at the index date.

**Results:**

Overall, 15,798 patients with ASCVD were included in the study (mean age: 71.1 years; 57% men), of whom 34% had CKD. The proportion of patients with systemic inflammation at the index date was 58% (65% among CKD patients) and 56% (62% among CKD patients) at the end of the study. Patients with systemic inflammation were more frequently smokers, obese, with comorbidities, and had higher low-density lipoprotein cholesterol and triglycerides levels than patients without systemic inflammation. Overall, patients with ASCVD and systemic inflammation used statins and aspirin less frequently compared to patients without systemic inflammation, while they used antibiotics, anticoagulants, and antihypertensives more frequently.

**Conclusion:**

Systemic inflammation prevalence is high among patients with ASCVD in Spain, especially among patients with comorbid CKD. Therapeutic strategies focused on targeting systemic inflammation may have beneficial effects in reducing the burden of ASCVD.

## Highlights

• Atherosclerotic cardiovascular disease (ASCVD) affects ∼10% of the Spanish population, contributing to 28% of all deaths.
• Despite optimal therapy, patients with ASCVD remain at high risk of recurrent major adverse cardiovascular events; systemic inflammation has been identified as an independent driver of residual cardiovascular risk.
• In Spain, systemic inflammation prevalence is high among patients with ASCVD, especially among patients with comorbid CKD.
• Patients with systemic inflammation also had higher prevalences of several comorbidities and atherogenic dyslipidaemia.
• This study highlights the need for novel strategies that target the reduction of inflammation for the secondary prevention of cardiovascular events and mortality in patients with ASCVD and systemic inflammation.

## Introduction

Atherosclerotic cardiovascular disease (ASCVD) is the leading cause of mortality in developed countries, posing a substantial burden in terms of morbidity and utilisation of healthcare resources (1). Globally, over half a billion people suffer from cardiovascular diseases, with nearly 20 million deaths annually (2), and approximately three-quarters of these cases are attributed to atherosclerotic diseases, particularly ischemic heart disease and stroke (2, 3). In Spain, ASCVD affects nearly 10% of the population, contributing to 28% of deaths and 13% of hospital admissions (4, 5).

Current guidelines for the management of patients with established ASCVD focus on lifestyle optimisation and more stringent control of classical modifiable cardiovascular risk factors, including diabetes, hypertension, and low-density lipoprotein cholesterol (LDL-C) (6). Despite optimal therapy, patients with ASCVD remain at high risk of recurrent major adverse cardiovascular events.

In recent years, there has been a growing recognition of systemic inflammation as an independent driver of residual cardiovascular risk (6–8). Increasing evidence has highlighted the pivotal role of systemic inflammation in the pathogenesis of ASCVD (9). Systemic inflammation can promote ASCVD progression during clinically stable phases, exacerbate the disease by advancing atherosclerosis, trigger atherosclerotic plaque destabilisation and acute coronary syndromes, and respond to cardiomyocyte necrosis in myocardial infarction (MI) (10). C-reactive protein (CRP) is a biomarker of systemic inflammation. In healthy women, high levels of CRP were predictive of incident cardiovascular events during a 30-year period (11). Systemic inflammation, defined by a CRP level equal to or greater than 2 mg/L, has been associated with a poor prognosis in patients with ASCVD, including an increased risk of cardiovascular events and higher overall mortality (7, 8, 12).

Chronic kidney disease (CKD) is associated with a state of systemic inflammation that contributes to faster renal deterioration and disease progression (13, 14) and is independently associated with an increased incidence of ASCVD and risk of MI and stroke (15–18).

Targeting systemic inflammation in patients with ASCVD, especially in those with comorbid CKD, may improve these high-risk patients' outcomes (19–23). Despite emerging evidence on the role of systemic inflammation as a risk factor in the progression of ASCVD (24–27), the epidemiology of systemic inflammation in patients with ASCVD within the general population has not been thoroughly investigated.

This study aims to generate real-world evidence to understand the prevalence and characteristics of systemic inflammation in patients with ASCVD and CKD in Spain, as it is crucial to better understand the residual cardiovascular risk, clinical characteristics, and unmet needs of patients with ASCVD in the real-world setting to inform healthcare providers and deliver optimised patient care to prevent recurrent cardiovascular events and mortality.

The specific objectives of the study are as follows: (1) to estimate the prevalence of systemic inflammation in patients with ASCVD (overall and by CKD status); (2) to describe the characteristics of patients with ASCVD by systemic inflammation status, including demographics, clinical parameters, comorbidities, and medication use.

## Methods

### Study design and data source

This was a population-based, retrospective, observational cohort study. Data were collected from the Spanish arm of The Health Improvement Network (THIN®) database (https://www.the-health-improvement-network.com/). THIN® is a secondary, irreversible anonymised database from primary care and ambulatory specialist care, linked at the patient level, with data recorded by general practitioners (GPs) during routine clinical practice. In Spain, THIN® data are routinely derived from electronic health records and include data on patient characteristics, prescriber information, and patient medical history, including clinical diagnostics (diagnosis recorded using the international classification of diseases, version 9: ICD-9), prescriptions (drugs and procedures), and laboratory test results. THIN® holds clinical data for ∼3 million individuals living in Spain, including approximately 1.2 million active patients with at least one visit during the previous 12 months (i.e., ∼3% of the Spanish general population). The database is updated monthly, with 1–3 months of lag time for the availability of the most recent data.

This study was carried out following the recommendations for clinical investigations included in the Declaration of Helsinki of the World Medical Association. The data used in this study are derived from a secondary database, where patients had previously provided informed consent at the source before the irreversible anonymisation of the database. For the processing and transfer of personal data to the THIN® Spain database, informed and unequivocal consent is obtained from both patients and their clinical practices. If any individual exercises their right to revoke consent for data processing, the patient's information is removed from the database prior to anonymisation. Since the database was anonymised at its origin and prior informed consent was ensured, ethical approval was not required for this study.

### Study population

Adult patients (age ≥18 years) with ASCVD and at least one eligible CRP measurement recorded between 1 January 2014 and 31 July 2023 (latest data available at the time of data extraction) were included in the study (Supplementary Table S1). ASCVD was defined by ICD-9 records for atherosclerotic coronary events (e.g., MI, angina, other ischemic conditions, or undergoing revascularisation), cerebrovascular events (e.g., occlusion of cerebral arteries or transient cerebral ischemia), peripheral arterial disease, and other atherosclerotic vascular diseases (e.g., generalised and unspecified atherosclerosis), as presented in the Appendix. Patients without an eligible CRP measurement were excluded from the study.

### Key variables

Systemic inflammation was defined as a CRP measurement ≥2 mg/L (10). CRP measurements were not considered eligible for the present study and excluded if they were recorded: (i) before first diagnosis of ASCVD; (ii) concomitantly with active infections (i.e., all CRP measurements >20 mg/L; CRP test taken within 2 months after or 7 days before antibiotic/antiviral prescriptions); (iii) under the effect of immunosuppressants (i.e., CRP tests taken less than 3 months after the prescription of immunosuppressant drugs); (iv) in patients with active cancer (i.e., CRP test taken less than 3 years after a diagnosis code for a malignancy, except non-melanoma skin cancer). In the case of multiple eligible CRP measurements taken within 3 months, they were grouped and averaged using a geometric mean. Notably, information about the type of CRP testing (standard or high-sensitivity) is not recorded in the THIN® database.

CKD was defined as a prior record of stage 3 or higher CKD diagnosis, estimated glomerular filtration rate [eGFR, calculated using the Chronic Kidney Disease Epidemiology Collaboration (CKD-EPI) 2021 formula] <60 ml/min/1.73 m^2^, or a urinary albumin-to-creatinine ratio (UACR) ≥ 30 mg/g (28, 29). Patients' comorbidities were defined using primary care diagnoses, and drug prescriptions were classified using the anatomical therapeutic chemical (ATC) classification system (codes provided in the Appendix).

### Statistical analyses

Categorical variables were reported using percentages and continuous variables were reported using mean ± standard deviation or median [interquartile range] according to a normal or skewed distribution, respectively.

The demographic [e.g., sex, age, smoking status, and socioeconomic status (SES)] and clinical characteristics of the patients were those reported at the time of the first eligible CRP measurement, which was set as the index date, or the most recent information available prior to the index date. Low SES was defined as residents with minimum earnings, a drug reimbursement rate of 100%, or non-retired patients with a drug reimbursement rate ≥90%.

For laboratory measurements [i.e., eGFR, UACR, haemoglobin level, total cholesterol, high-density lipoprotein cholesterol (HDL-C), LDL-C, and triglycerides] and clinical measurements [body mass index (BMI), systolic blood pressure (SBP), and diastolic blood pressure (DBP)], the most recent information available within 18 months before the index date was considered. The history of comorbidities was defined using the presence of ICD-9 diagnosis codes recorded at any time before the index date. The use of medications was defined by dispensation with selected ATC codes in the 12 months before the index date. The dispensation of medications in the 12 months after the index date was also reported.

The proportion of systemic inflammation in patients with ASCVD was assessed at three different time points:

(i) at first eligible CRP measurement, i.e., the number of patients with first eligible CRP measurement ≥ 2 mg/L divided by the total number of patients included in the study;
(ii) at the latest available date (point prevalence), i.e., the proportion of patients with systemic inflammation among the patients alive at the data cutoff date (31 July 2023) and with at least one eligible CRP measurement in the prior 18 months;
(iii) over the entire study period, i.e., the proportion of patients with systemic inflammation at any time during the study period among the overall study population.

A diagram illustrating how patients were defined in terms of systemic inflammation at each time point is shown in Supplementary Figure S2. Analyses were conducted for all patients with ASCVD included in the study and separately for the subgroup of patients with ASCVD and CKD at first CRP measurement. Within the patient population with CKD, the proportion of patients with systemic inflammation was further assessed by CKD stage.

### Sensitivity analysis

As systemic inflammation is elevated with chronic inflammatory conditions, the proportion of patients with systemic inflammation was further assessed after the exclusion of patients with ASCVD and comorbid inflammatory and rheumatoid diseases, as defined in the Appendix.

## Results

### Study population

**Figure 1:**
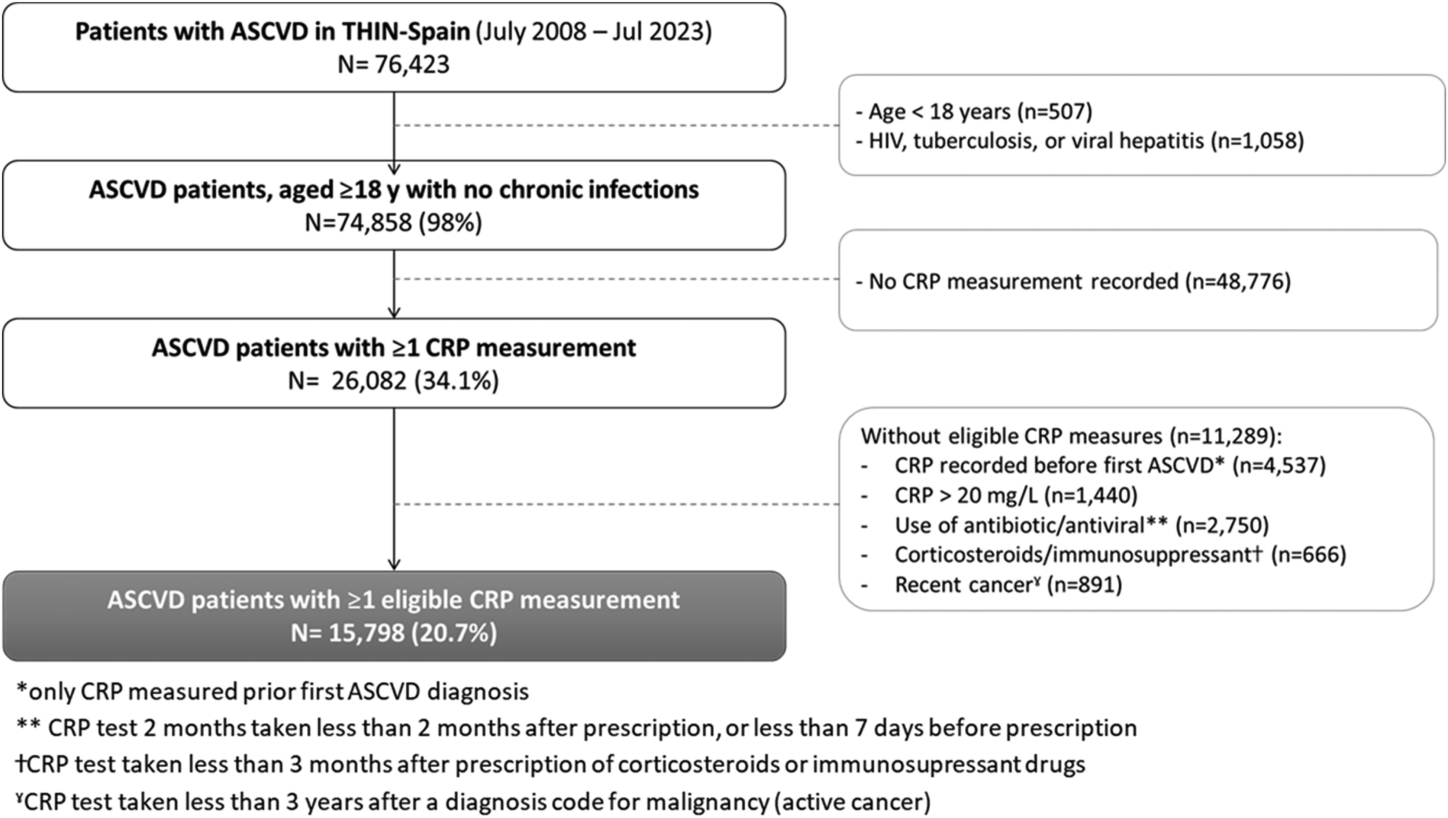
Flowchart of the study population.

Overall, 76,423 patients with a primary care diagnosis of ASCVD between 1 January 2014 and 31 July 2023 were identified in the THIN-Spain database (Figure 1). After the exclusion of patients aged <18 years or with chronic infection diseases, 34% of the adult patients had at least one CRP measurement during the study period (*n* = 26,082) of whom *n* = 15,798 had at least one eligible CRP measurement and were included in the analyses. Overall, 32% (*n* = 5,111) of the included patients with ASCVD had CKD diagnosed at the time of their first eligible CRP measurement, 62% (*n* = 9,712) had no prior diagnosis of CKD, and a CKD status was not assigned in 6% of patients who did not have any eGFR measurement or diagnoses of CKD. A majority of the included patients had a diagnosis of coronary artery disease (54.5%), 42.3% had been diagnosed with a cerebrovascular atherosclerotic event, 7.7% had peripheral artery disease (PAD), and 9.9% suffered from other atherosclerotic vascular diseases.

### Prevalence of systemic inflammation in ASCVD

**Figure 2:**
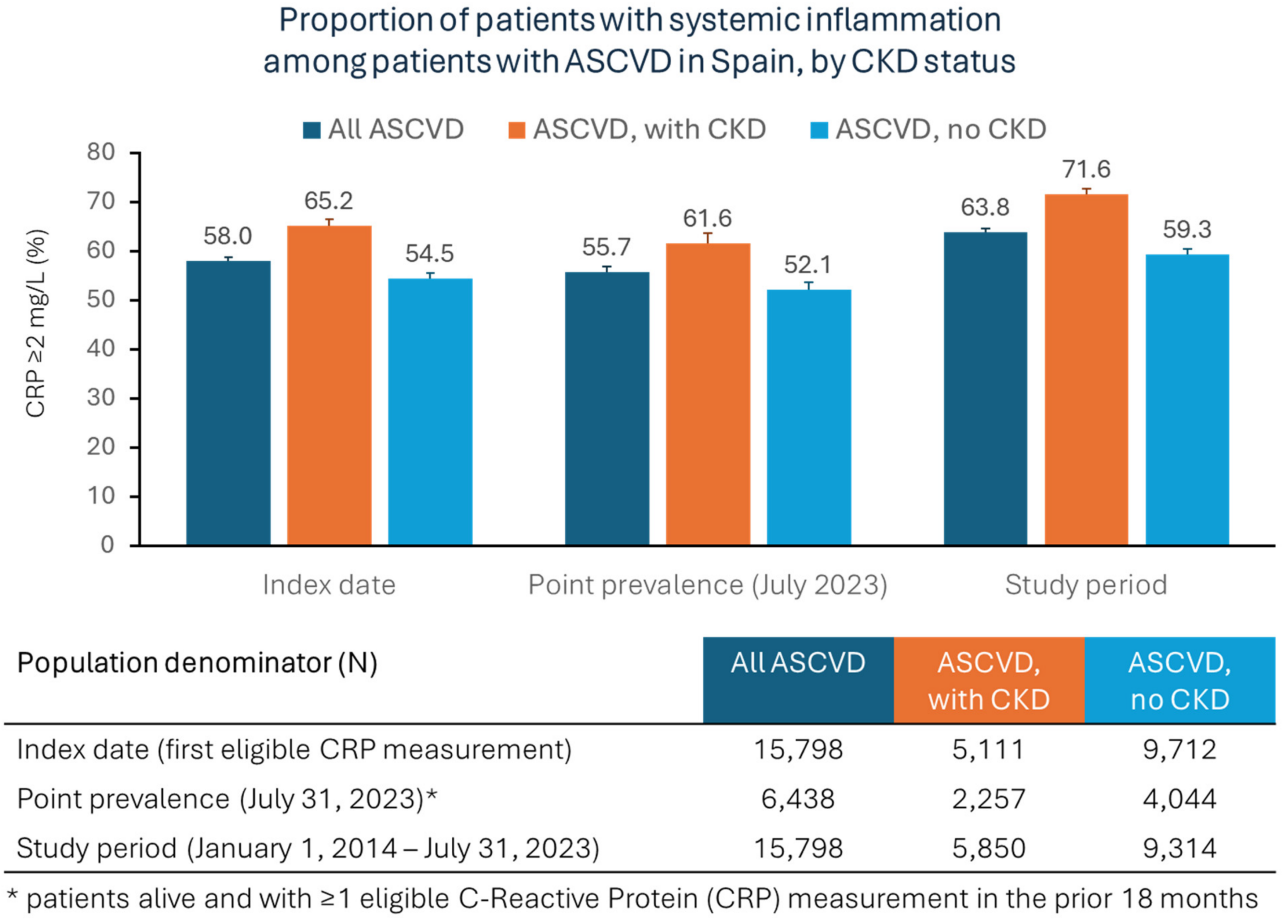
Prevalence of systemic inflammation among patients with ASCVD in Spain, overall and by CKD status (central illustration).

Most of the patients (67%) had only one eligible CRP measurement during the study period, while 33% had two or more measurements. The mean and median values of eligible CRP measurements were 3.7 mg/L (standard deviation: 3.8) and 2.4 mg/L (interquartile range: 1.2–4.8), respectively. Overall, 58% of the patients with ASCVD had systemic inflammation at their first eligible CRP measurement (Figure 2). The proportion was higher in the patients with CKD compared to the patients without CKD (65% vs. 55%, respectively). The point prevalence of systemic inflammation at the data cutoff date (31 July 2023) was 56% among alive patients with at least one CRP measurement in the prior 18 months, and it was greater among the patients with ASCVD and CKD (62%) than in the patients with ASCVD without CKD (52%). Overall, 64% of the patients had at least 1 CRP measurement greater than 2 mg/L during the entire study period. Among the patients with ASCVD and CKD, the proportion of patients with systemic inflammation was greater among the patients with stage 4 CKD or stage 5 CKD compared to patients with a lower CKD stage (Supplementary Table S1).

The proportion of patients with systemic inflammation at the index date was slightly higher in women than men (59% vs. 57%, respectively), both among all patients with ASCVD and among patients with comorbid CKD (Supplementary Figure S3). Overall, patients with ASCVD older than 75 years had a higher proportion of systemic inflammation than younger age groups at the index date (56%, 57%, and 60% among patients aged <65, 65–74, and ≥75 years, respectively). In contrast, among the patients with ASCVD and comorbid CKD, the proportion of systemic inflammation was higher in the patients <65 years old than in older age groups (69%, 64%, and 65% among patients aged <65, 65–74, and ≥75 years, respectively) (Supplementary Figure S4).

### Demographic and clinical characteristics

The mean age of the overall ASCVD study population was 71 years and 57% were men (Table 1). Compared to the patients without systemic inflammation, the patients with systemic inflammation were older, included more women and smokers and had a lower SES and higher BMIs. The patients with systemic inflammation had a higher prevalence of CKD and other comorbidities including diabetes, hypertension, PAD, heart failure, chronic obstructive pulmonary disease (COPD), and atrial fibrillation. Furthermore, the patients with systemic inflammation had lower baseline eGFR, haemoglobin, and HDL-C levels and higher LDL-C and triglycerides levels than the patients without systemic inflammation.

**Table 1:** Demographic and clinical characteristics of patients with ASCVD by systemic inflammation (SI) status. Data expressed as mean ± standard deviation, median [IQR], or percentage.

Variable	ASCVD, overall	ASCVD with SI	ASCVD without SI	*p*-value
	*N* = 15,798	*N* = 9,169	*N* = 6,629	
Age (years)	71.19 ± 12.11	71.65 ± 12.11	70.54 ± 12.08	**<0** **.** **001**
Female sex	42.90%	43.72%	41.77%	**0** **.** **014**
Current smoker	22.33%	24.53%	19.29%	**<0** **.** **001**
Low SES	15.48%	16.42%	14.18%	**<0** **.** **001**
BMI (kg/m^2^)	29.37 ± 5.27	30.25 ± 5.5	28.1 ± 4.63	**<0** **.** **001**
SBP (mmHg)	136.21 ± 29.74	136.56 ± 29.36	135.72 ± 30.28	0.177
DBP (mmHg)	79.38 ± 126.85	77.55 ± 13.81	81.98 ± 196.24	0.156
CRP (mg/L)	2.4 [1.2, 4.9]	4.3 [2.9, 7.4]	1.1 [0.7, 1.5]	**<0** **.** **001**
eGFR (mL/min/1.73 m^2^)	70.9 ± 20.7	69.2 ± 21.3	73.3 ± 19.5	**<0** **.** **001**
UACR (mg/g)	15.7 [7.5, 45.9]	17.5 [8.1, 52.5]	13.4 [6.8, 36.6]	**<0** **.** **001**
Haemoglobin (g/dL)	13.43 ± 2.17	13.32 ± 2.18	13.59 ± 2.15	**<0** **.** **001**
Total cholesterol (mg/dL)	173.67 ± 43.29	176.03 ± 44.48	170.51 ± 41.45	**<0** **.** **001**
HDL-C (mg/dL)	47.63 ± 13.11	46.03 ± 12.57	49.76 ± 13.51	**<0** **.** **001**
LDL-C (mg/dL)	99.55 ± 35.9	101.97 ± 36.72	96.42 ± 34.56	**<0** **.** **001**
Triglycerides (mg/dL)	135.47 ± 87.29	143.83 ± 92.9	124.27 ± 77.77	**<0** **.** **001**
ASCVD type				
Coronary disease	54.5%	53.2%	56.2%	**<0** **.** **001**
Cerebrovascular disease	42.3%	42.7%	41.7%	0.202
PAD	7.7%	9.0%	5.8%	**<0** **.** **001**
Other	9.9%	11.1%	8.3%	**<0** **.** **001**
Comorbidities				
CKD	32.4%	36.4%	26.8%	**<0** **.** **001**
CKD stage				
Stage 5	0.4%	0.5%	0.2%	
Stage 4	3.4%	4.2%	2.4%	
Stage 3	24.8%	27.5%	21.0%	
Stage 1/2^a^	71.4%	67.8%	76.3%	
Unknown	0.1%	0.1%	0.1%	
Diabetes	34.3%	35.9%	32.1%	**<0** **.** **001**
Hypertension	65.6%	68.2%	62.1%	**<0** **.** **001**
COPD	29.6%	32.6%	25.3%	**<0** **.** **001**
Cancer^b^	1.5%	1.7%	1.2%	**0** **.** **018**
Dementia	5.3%	5.7%	4.6%	**0** **.** **004**
Heart failure	10.0%	12.0%	7.2%	**<0** **.** **001**
Atrial fibrillation	13.4%	15.0%	11.2%	**<0** **.** **001**
IBD	0.8%	0.9%	0.6%	**0** **.** **033**
Rheumatoid disease	18.3%	20.6%	15.1%	**<0** **.** **001**
CNS inflammatory disease	0.2%	0.2%	0.1%	0.204
Liver disease	5.0%	5.6%	4.0%	**<0** **.** **001**

ASCVD, atherosclerotic cardiovascular disease; CKD, chronic kidney failure; SI, systemic inflammation; SES, socioeconomic status; SBP, systolic blood pressure; DBG, diastolic blood pressure; BMI, body mass index; CRP, C-reactive protein; eGFR, estimated glomerular filtration rate; UACR, urinary albumin-to-creatinine ratio; HDL-C, high-density lipoprotein cholesterol; LDL-C, low-density lipoprotein cholesterol; COPD, chronic obstructive pulmonary disease; IBD, inflammatory bowel disease; CNS, central nervous system.

Statistically significant *p*-values (*p* < 0.05) are reported in bold.

^a^ CKD stage 1/2: patients with eGFR ≥ 60 ml/min/1.73 m^2^ and UACR ≥ 30 mg/g.

^b^ Cancer (excluding non-melanoma skin cancer) recorded >3 years before the first eligible CRP measurement.

Among the patients with ASCVD and CKD, those with systemic inflammation were more frequently smokers and had higher BMIs; lower eGFR, haemoglobin, and HDL-C levels; and higher levels of LDL-C and triglycerides. The patients with ASCVD, CKD, and inflammation had a higher prevalence of COPD, heart failure, atrial fibrillation, rheumatoid disease, and PAD than the patients with ASCVD and CKD but without systemic inflammation (Table 2).

**Table 2:** Demographic and clinical characteristics of patients with ASCVD with CKD by SI status. Data expressed as mean ± standard deviation, median [IQR], or percentage.

Variable	ASCVD + CKD, overall	ASCVD + CKD, With SI	ASCVD + CKD, Without SI	*p*-value
	*N* = 5,111	*N* = 3,334	*N* = 1,777	
Age (years)	77.53 ± 9.74	77.52 ± 9.9	77.55 ± 9.43	0.915
Female sex	42.6%	43.0%	41.9%	0.454
Current smoker	16.6%	17.9%	14.1%	**<0** **.** **001**
Low SES	17.4%	17.6%	17.2%	0.735
BMI (kg/m^2^)	29.48 ± 5.3	30.17 ± 5.55	28.24 ± 4.59	**<0** **.** **001**
SBP (mmHg)	137.2 ± 29.11	136.99 ± 32.06	137.6 ± 22.41	0.522
DBP (mmHg)	77.61 ± 145.1	74.91 ± 13.33	82.78 ± 247.09	0.282
CRP (mg/L)	2.9 [1.5, 5.9]	4.6 [3.0, 7.8]	1.1 [0.8, 1.5]	**<0** **.** **001**
eGFR (mL/min/1.73 m^2^)	50.43 ± 17.58	49.89 ± 17.76	51.43 ± 17.21	**<0** **.** **001**
UACR (mg/g)	46.7 [20.4, 123.8]	49.0 [20.8, 125.4]	43.3 [19.7, 116.7]	**0** **.** **066**
Haemoglobin (g/dL)	12.8 ± 2.22	12.75 ± 2.2	12.89 ± 2.25	**0** **.** **033**
Total cholesterol (mg/dL)	167.81 ± 42.82	169.79 ± 44.3	164.2 ± 39.74	**<0** **.** **001**
HDL-C (mg/dL)	44.99 ± 12.36	43.79 ± 12.07	47.14 ± 12.58	**<0** **.** **001**
LDL-C (mg/dL)	94.31 ± 34.99	96.5 ± 35.84	90.45 ± 33.09	**<0** **.** **001**
Triglycerides (mg/dL)	146.94 ± 91.69	152.91 ± 98.44	136.1 ± 76.82	**<0** **.** **001**
ASCVD type				
Coronary disease	56.0%	55.3%	57.3%	0.175
Cerebrovascular disease	42.3%	42.2%	42.4%	0.921
PAD	10.8%	11.6%	9.1%	**0** **.** **006**
Other	11.9%	13.0%	9.8%	**0** **.** **001**
Comorbidities				
CKD	100%	100%	100%	–
CKD stage				**<0** **.** **001**
Stage 5	1.1%	1.2%	0.9%	
Stage 4	9.8%	10.7%	8.2%	
Stage 3	71.1%	70.4%	72.3%	
Stage 1/2^a^	18.0%	17.7%	18.6%	
Unknown	0.0%	0.0%	0.0%	
Diabetes	49.6%	49.5%	49.8%	0.831
Hypertension	79.0%	79.6%	77.9%	0.172
COPD	33.5%	35.9%	28.9%	**<0** **.** **001**
Cancer^b^	2.2%	2.3%	2.0%	0.469
Dementia	7.8%	8.2%	7.0%	0.133
Heart failure	19.3%	21.8%	14.6%	**<0** **.** **001**
Atrial fibrillation	21.6%	22.7%	19.4%	**0** **.** **006**
IBD	0.7%	0.7%	0.7%	0.942
Rheumatoid disease	28.3%	30.4%	24.4%	**<0** **.** **001**
CNS inflammatory disease	0.2%	0.2%	0.2%	0.928
Liver disease	4.7%	5.1%	3.9%	0.055

ASCVD, atherosclerotic cardiovascular disease; CKD, chronic kidney failure; SI, systemic inflammation; SES, socio-economic status; SBP, systolic blood pressure; DBG, diastolic blood pressure; BMI, body mass index; CRP, C-reactive protein; eGFR, estimated glomerular filtration rate; UACR, urinary albumin-to-creatinine ratio; HDL-C, high-density lipoprotein cholesterol; LDL-C, low-density lipoprotein cholesterol; COPD, chronic obstructive pulmonary disease; IBD, inflammatory bowel disease; CNS, central nervous system.

Statistically significant *p*-values (*p* < 0.05) are reported in bold.

^a^ CKD stage 1/2: patients with eGFR ≥ 60 ml/min/1.73 m^2^ and UACR ≥ 30 mg/g.

^b^ Cancer (excluding non-melanoma skin cancer) recorded >3 years before the first eligible CRP measurement.

### Treatment utilisation

Drug utilisation in the 12 months prior to the first eligible CRP measurement in patients with ASCVD is summarised in Table 3. Compared to the patients without systemic inflammation, those with systemic inflammation generally had a greater utilisation of most medications, including angiotensin-converting enzyme inhibitors/angiotensin II receptor blockers (ACEis/ARBs), mineralocorticoid receptor antagonists (MRAs), diuretics and anticoagulants, except for aspirin and antiplatelet agents, statins, and other lipid-lowering agents that were less prescribed in the patients with systemic inflammation.

**Table 3:** Drug utilisation in patients with ASCVD, by SI status. The table reports the percentage of patients using each pharmacological group in the 12 months prior to the first eligible CRP measurement.

Drug utilisation	All ASCVD	ASCVD with SI	ASCVD without SI	*p*-value
	*N* = 15,798	*N* = 9,169	*N* = 6,629	
Corticosteroids	6.1%	6.6%	5.5%	**0** **.** **003**
Immunosuppressants	0.4%	0.5%	0.3%	0.070
Antibiotics, antivirals, and antimycotics	34.9%	37.0%	31.9%	**<0** **.** **001**
Antibiotics	33.8%	36.1%	30.7%	**<0** **.** **001**
Antivirals	1.1%	1.1%	1.1%	0.999
Antimycotics	1.0%	1.0%	1.0%	0.908
Antiplatelet agents	63.7%	62.4%	65.6%	**<0** **.** **001**
Aspirin	53.9%	52.6%	55.7%	**<0** **.** **001**
Anticoagulants (VKAs and DOACs)	13.5%	15.2%	11.3%	**<0** **.** **001**
NSAIDs	31.1%	31.6%	30.3%	0.075
ACEi/ARBs	59.6%	61.3%	57.2%	**<0** **.** **001**
MRAs	5.1%	5.6%	4.3%	**<0** **.** **001**
Beta-blockers	42.4%	42.7%	41.9%	0.305
Diuretics	24.6%	27.7%	20.2%	**<0** **.** **001**
Calcium channel Blockers	19.6%	20.5%	18.3%	**0** **.** **001**
Glucose-lowering agents	29.4%	30.6%	27.8%	**<0** **.** **001**
SGLT2 inhibitors	3.7%	4.0%	3.3%	**0** **.** **031**
GLP-1 receptor agonists	1.6%	1.7%	1.3%	**0** **.** **037**
Statins	68.5%	65.3%	72.8%	**<0** **.** **001**
Other lipid-lowering agents	3.2%	3.0%	3.5%	**0** **.** **046**
Ezetimibe	2.9%	2.7%	3.2%	**0** **.** **035**
PCSK9 inhibitors	0.3%	0.2%	0.3%	0.569
Omega 3	0.2%	0.2%	0.1%	0.447
Fibrates, resins, and nicotinic acid	4.8%	5.1%	4.3%	**0** **.** **012**
Other blood pressure medications	3.7%	4.0%	3.3%	**0** **.** **026**
Colchicine	1.7%	1.9%	1.4%	**0** **.** **027**

ASCVD, atherosclerotic cardiovascular disease; CKD, chronic kidney disease; SI, systemic inflammation; VKA, vitamin K antagonists; DOAC, direct oral anticoagulant; NSAID, non-steroid anti-inflammatory drugs; ACEi, angiotensin-converting enzyme inhibitor; ARB, angiotensin II receptor blocker; MRA, mineralocorticoid receptor antagonist; SGLT2, sodium-glucose cotransporter-2; GLP-1, glucagon-like peptide-1; PCSK9, proprotein convertase subtilisin/kexin type-9.

Statistically significant p-values (*p* < 0.05) are reported in bold.

Among the patients with ASCVD and CKD, those with systemic inflammation used more antibiotics, anticoagulants, MRAs, and diuretics but they had a lower utilisation of antiplatelet agents (including aspirin) and statins in the 12 months before the index date than the patients without systemic inflammation (Table 4). Similar drug utilisation was observed during the 12 months after the index date in patients with ASCVD overall and in those with comorbid CKD (Supplementary Tables S2 and S3).

**Table 4:** Drug utilisation in patients with ASCVD with CKD, by SI status. The table reports the percentage of patients using each pharmacological group in the 12 months prior to the first eligible CRP measurement.

Drug utilisation	All ASCVD + CKD	ASCVD + CKD, with SI	ASCVD + CKD, without SI	*p*-value
	*N* = 5,111	*N* = 3,334	*N* = 1,777	
Corticosteroids	6.9%	7.1%	6.5%	0.392
Immunosuppressants	0.4%	0.4%	0.3%	0.533
Antibiotics, antivirals, and antimycotics	39.9%	41.8%	36.1%	**<0** **.** **001**
Antibiotics	39.1%	41.1%	35.5%	**<0** **.** **001**
Antivirals	1.1%	1.0%	1.3%	0.319
Antimycotics	0.9%	0.9%	0.8%	0.839
Antiplatelet agents	64.7%	63.5%	66.8%	**0** **.** **020**
Aspirin	53.1%	52.0%	55.0%	**0** **.** **041**
Anticoagulants (VKAs and DOACs)	21.2%	22.7%	18.5%	**<0** **.** **001**
NSAIDs	25.8%	26.1%	25.3%	0.520
ACEi/ARBs	71.4%	71.1%	72.1%	0.450
MRAs	8.7%	9.4%	7.4%	**0** **.** **020**
Beta-blockers	50.4%	50.2%	50.6%	0.782
Diuretics	41.1%	43.9%	35.8%	**<0** **.** **001**
Calcium channel blockers	26.2%	26.8%	24.9%	0.144
Glucose-lowering agents	43.1%	42.7%	44.0%	0.394
SGLT2 inhibitors	5.5%	5.5%	5.6%	0.938
GLP-1 receptor agonists	2.4%	2.6%	2.2%	0.396
Statins	71.2%	68.4%	76.4%	**<0** **.** **001**
Other lipid-lowering agents	3.2%	3.1%	3.3%	0.734
Ezetimibe	2.8%	2.7%	2.9%	0.723
PCSK9 inhibitors	0.2%	0.2%	0.2%	0.911
Omega 3	0.3%	0.3%	0.2%	0.626
Fibrates, resins, and nicotinic acid	6.8%	7.0%	6.6%	0.613
Other blood pressure medications	6.9%	7.0%	6.9%	0.961
Colchicine	2.3%	2.6%	1.9%	0.091

ASCVD, atherosclerotic cardiovascular disease; CKD, chronic kidney disease; SI, systemic inflammation; VKA, vitamin K antagonists; DOAC, direct oral anticoagulant; NSAID, non-steroid anti-inflammatory drugs; ACEi, angiotensin-converting enzyme inhibitor; ARB, angiotensin II receptor blocker; MRA, mineralocorticoid receptor antagonist; SGLT2, sodium-glucose cotransporter-2; GLP-1, glucagon-like peptide-1; PCSK9, proprotein convertase subtilisin/kexin type-9.

Statistically significant *p*-values (*p* < 0.05) are reported in bold.

## Discussion

### Summary findings

This study investigated the prevalence of systemic inflammation in patients with ASCVD using electronic medical records from a nationwide, population-based primary care database in Spain and reported the demographic and clinical characteristics of the patients. The main findings of the study are as follows:

• In Spain, 56% of patients with ASCVD had systemic inflammation, and the proportion increased to 62% among those with comorbid CKD.
• Systemic inflammation was more frequent in women; smokers; patients with obesity and comorbidities, including PAD, hypertension, diabetes, heart failure, atrial fibrillation, and non-cardiovascular comorbidities; and patients with a more atherogenic risk profile (higher LDL-C and triglyceride levels and lower HDL-C level).
• The patients with ASCVD and systemic inflammation used statins and aspirin less frequently and antibiotics, anticoagulants, MRAs, and diuretics more frequently compared to the patients without systemic inflammation.

The analyses highlighted that most of the Spanish patients with ASCVD had evidence of systemic inflammation, and the proportion was 62% among the patients with comorbid CKD. Considering that nearly 5 million patients in Spain live with ASCVD (5), our estimates suggest that nearly 2.5 million patients with ASCVD have systemic inflammation, of whom approximately 1 million have comorbid CKD.

Systemic inflammation has been increasingly recognised as a significant risk factor for cardiovascular events. Clinical trials have shown that anti-inflammatory strategies may reduce the incidence of cardiovascular events in patients with ASCVD, underscoring the critical role of inflammation in residual cardiovascular risk (30–32). The phase-3 ZEUS trial is currently testing whether targeting systemic inflammation with ziltivekimab, a monoclonal antibody that targets interleukin-6, can reduce the risk of recurrent cardiovascular events in patients with established ASCVD, CKD, and systemic inflammation (33).

This is one of the first studies investigating the prevalence of systemic inflammation in patients with ASCVD from the general population, and the first one carried out in Spain. To our knowledge, only two other studies have reported the prevalence of systemic inflammation in representative samples of patients with ASCVD. A recent study using the Stockholm's healthcare database in Sweden showed that 59% of adult patients with ASCVD and 69% of patients with ASCVD and CKD had systemic inflammation at their first high-sensitivity CRP measurement (12). In the US, a study using the National Health and Nutrition Examination Survey (NHANES) data reported that 55% of adult patients with ASCVD and 62% of patients with ASCVD and stage 3 or 4 CKD had systemic inflammation (34). These estimates for systemic inflammation among the ASCVD populations in Sweden and the US are largely aligned with our findings in the THIN-Spain database. Another recent publication on ∼24,000 participants in three randomised, placebo-controlled phase-3 trials investigating the efficacy of semaglutide (SELECT, SOUL, and FLOW) reported that systemic inflammation was present in 56% of patients with ASCVD and CKD, as compared to 47% of patients with ASCVD and without CKD (35).

CRP is currently the most relevant biomarker for the assessment of residual inflammatory risk (36), and a cutoff of 2 mg/L has been adopted in several clinical trials, including CANTOS, JUPITER, RESCUE, and ZEUS (21, 33, 37, 38) to identify a subgroup of patients with ASCVD at greater risk of atheroprogression and mortality (7).

Systemic inflammation was more prevalent in patients with ASCVD and CKD, with greater prevalence at more advanced stages of the disease. In fact, CKD is associated with a proinflammatory milieu (17). Furthermore, inflammation is a critical factor in the progression of CKD, and persistent systemic inflammation has been associated with a faster decline of renal function and hypertension and increased mortality and cardiovascular events in this population (14).

In our study, the patients with ASCVD and systemic inflammation also had a greater prevalence of several comorbidities (including higher BMI, hypertension, diabetes, PAD, heart failure, and atrial fibrillation) than the patients without systemic inflammation. This finding is in line with current medical literature, as systemic inflammation has been reported to play a role in the onset and progression of several diseases such as diabetes (39), hypertension (40), Alzheimer's disease and dementia (41, 42), atrial fibrillation (43), rheumatoid arthritis (44), and liver complications such as non-alcoholic fatty liver disease and cirrhosis (45). CRP, moreover, has been identified as a physiological marker of multimorbidity (46), suggesting that systemic inflammation may negatively affect multiple organs simultaneously and increase the risk of comorbidities.

In our study, greater proportions of smokers were observed among the patients with ASCVD and systemic inflammation compared to those without systemic inflammation. The association of tobacco smoking with increased levels of inflammation markers, including CRP, is well-acknowledged (47).

The patients with ASCVD and systemic inflammation had higher BMIs compared to those without systemic inflammation. The link between obesity and systemic chronic inflammation is well-established (48). Adipose tissue can activate the innate immune system and the release of inflammatory cytokines, sustaining systemic inflammation and causing an increase in CRP levels; in turn, sustained systemic inflammation may induce tissue fibrosis and necrosis and ultimately cause significant organ damage (48). Atherogenic dyslipidaemia, which is another independent risk factor for future cardiovascular events, has been observed in a higher proportion of patients with ASCVD and systemic inflammation (49). In patients with ASCVD treated with statins, however, systemic inflammation was a greater driver of recurrent cardiovascular events than increased LDL-C levels (19).

In our study, patients with ASCVD and CRP ≥ 2 mg/L more frequently used drugs such as antibiotics, oral anticoagulants, MRAs, and diuretics than patients with lower CRP levels. This likely reflects worse health conditions and a greater proportion of comorbidities in patients with systemic inflammation (46). In contrast, patients with ASCVD but without systemic inflammation more frequently used aspirin and statins compared to those with systemic inflammation. The lower utilisation of aspirin may be due to the increased use of anticoagulants in the latter. However, the reduced use of lipid-lowering therapies, particularly statins, in patients with ASCVD and systemic inflammation is difficult to explain, especially given their generally more atherogenic profile. As a result, those patients with higher cardiovascular risk may not be achieving strict target lipid levels in secondary prevention.

The use of medications to control classical cardiovascular risk factors (e.g., ACEi/ARBs, statins and lipid-lowering therapies) in patients with ASCVD, remains suboptimal, both in patients with or without systemic inflammation and with or without CKD. Furthermore, mean LDL-C levels were significantly higher than the values recommended by the current guidelines (50), suggesting there is room for stricter control of known cardiovascular risk factors and further improvements in preventing recurrent ASCVD outcomes.

The strengths of this study include the use of a large population-based and nationwide sample: over 15,000 patients with ASCVD were characterised for CRP levels, demographic and clinical characteristics, treatment patterns, and a set of biomarkers routinely monitored in clinical practice. However, a limitation of this study was that the data analysis was restricted to information routinely documented in the THIN-Spain database. Inpatient diagnoses and procedures are not recorded in the THIN-Spain database, which may result in lower sensitivity in detecting ASCVD. Incomplete or erroneous documentation of diagnoses, prescriptions, and comorbidities cannot be entirely excluded since there was no case ascertainment through medical records. Another limitation of this study is that the THIN-Spain database did not record whether the CRP tests were performed using regular or high-sensitivity assays. This, however, may have impacted the results to a minor extent, as high-sensitivity CRP tests can measure CRP levels in the low-sensitivity range (i.e., below 1 mg/L) and do not denote a more specific differential diagnosis of systemic inflammation (51). Moreover, the proportion of patients with systemic inflammation in our study was similar to the proportions observed in the studies conducted using the Stockholm's healthcare database and NHANES data, in which all CRP assays were performed using high-sensitivity CRP methods (12, 34). Some CRP measurements may be affected by underlying infections or other inflammatory chronic conditions. To minimise this type of confounding, patients with chronic infections (e.g., HIV, tuberculosis, and viral hepatitis) and CRP measurements taken within 3 months of an acute infection diagnosis, the use of antibiotics or antivirals, concomitantly with immunosuppressants, or in patients with a recent cancer diagnosis were excluded from the analyses. A sensitivity analysis was performed by excluding patients with chronic inflammatory diseases, which gave a similar prevalence of systemic inflammation as the main analysis (Supplementary Table S4). However, residual confounding due to undiagnosed infections or inflammatory diseases may still be present.

Another limitation is that CRP measurement is not routinely performed in clinical practice, and only a subgroup of patients with ASCVD had systemic inflammation tested in our sample. In fact, despite systemic inflammation being acknowledged as a risk factor for incident cardiovascular events, only in the 2024 ESC guidelines is CRP testing recommended for risk stratification in secondary cardiovascular prevention (6). Without clinically available targeted treatments for systemic inflammation, the diagnosis of systemic inflammation does not currently lead to optimal management and improved outcomes in patients with ASCVD. As such, the demand for CRP measurements from cardiologists, nephrologists, and general practitioners is currently low. In the THIN-Spain database, only one-third of patients with diagnosed ASCVD had at least one CRP measurement performed during the study period, and only one-fifth had an eligible measurement after the exclusion of CRP measurements taken during concurrent acute or chronic infections, concomitantly with the use of immunosuppressants, or after a diagnosis of cancer. This may limit the generalisability of our estimates to the entire ASCVD population, as the characteristics of patients with CRP measurements may be different compared to patients with ASCVD for whom a CRP measurement was not available. In the THIN-Spain database, the patients with ASCVD and CRP measurements had similar age and body mass indexes but different proportions of women, smokers, and concomitant CKD than the patients with ASCVD and without a CRP measurement (Supplementary Table S5).

Further, some variables, such as BMI, UACR, and laboratory values, were not recorded for all the patients included in the THIN® database, and therefore results might be biased by some selection (e.g., GPs might record weight more frequently in patients with weight issues compared to patients with regular weights).

Finally, when interpreting the results of this study, it should be considered that the study design did not allow us to assess causality or chronological order in the link between systemic inflammation and comorbidities and concomitant treatments (e.g., if systemic inflammation occurs before or after the onset of the associated diseases), and the observed directions may have been affected by the presence of confounding factors.

## Conclusions

Systemic inflammation is highly prevalent among Spanish patients with ASCVD, particularly those with comorbid CKD, highlighting a subgroup of patients at increased risk of cardiovascular events and death despite statin therapy. However, most of the patients with ASCVD had never had their CRP levels assessed. Novel strategies that aim to reduce systemic inflammation may offer significant benefits for secondary prevention of cardiovascular events and mortality in a substantial proportion of patients with ASCVD and systemic inflammation. Routine CRP assessment in all ASCVD patients is crucial for identifying patients at higher risk and delivering optimal, targeted treatment.

## Data Availability

The data analyzed in this study is subject to the following licenses/restrictions: Data were collected from the Spanish arm of The Health Improvement Network (THIN®) database. Requests to access these datasets should be directed to https://www.the-health-improvement-network.com.

## Appendix

**Table A1:** Diagnosis codes for ASCVD.

ICD-9 code	Description
Coronary event	
410	Acute myocardial infarction
411	Ischemic heart disease
412	Old myocardial infarction
413	Angina pectoris
414	Chronic ischemic heart disease
Cerebrovascular event	
433	Occlusion and stenosis of precerebral arteries
434	Occlusion of cerebral arteries
435	Transient cerebral ischemia
436	Acute, but ill-defined, cerebrovascular disease
437	Other and ill-defined cerebrovascular disease
438	Late effects of cerebrovascular disease
Peripheral arterial disease (PAD)	
440.2	Atherosclerosis of native arteries of the extremities
440.3	Atherosclerosis of bypass graft of the extremities
440.4	Chronic total occlusion of artery of the extremities
443.9	Peripheral vascular disease, unspecified
Other atherosclerotic vascular diseases	
440	Atherosclerosis
440.0	Atherosclerosis of aorta
440.1	Atherosclerosis of the renal artery
440.8	Atherosclerosis of other specified arteries
440.9	Generalised and unspecified atherosclerosis
441	Aortic aneurysm and dissection
445	Atheroembolism

**Table A2:** Definition of chronic kidney disease (CKD).

CKD assessment	Definition	Description
CKD status		Diagnosis of CKD stage 3+, GFR < 60 mL/min/1.73 m^2^, or albuminuria (UACR = 30+)
Clinical diagnosis, ICD-9 codes	585	CKD
	585.1	CKD, stage 1
	585.2	CKD, stage 2
	585.3	CKD, stage 3
	585.4	CKD, stage 4
	585.5	CKD, stage 5
	585.6	End-stage renal disease
	585.9	CKD, unspecified
GFR measurement		GFR < 60 mL/min/1.73 m^2^
		GFR: 45–59 = stage 3a CKD
		GFR: 30–44 = stage 3b CKD
		GFR: 15–29 = stage 4 CKD
UACR		UACR ≥ 30 mg/g

**Table A3:** Exclusion criteria for CRP measurements.

Description	Exclusion criteria for CRP measurement
CRP measurement before ASCVD diagnosis	CRP measure dated before the first occurrence of ASCVD diagnosis
Active infection	CRP > 20 mg/L (= acute infection)
	Antibiotic/antimycotic/antiviral prescribed within 2 months before and 7 days after CRP measurement
Use of immunosuppressants	Corticosteroids (H02) and immunosuppressants (L04) administered within 2 months prior to CRP measurement
Recent cancer	Diagnosis of cancers (ICD-9 code: 140–209, except non-melanoma skin cancer: ICD-9 code: 173) within 3 years prior to the CRP measurement

**Table A4:** Definitions for comorbidities.

Comorbidity	ICD-9 diagnosis code	ATC code
Chronic infections (exclusion criteria for this study)	010–018 Tuberculosis	
	042–044 HIV	
	070 Viral hepatitis	
Diabetes	249 Secondary diabetes mellitus	A10 (3 + prescriptions/year)
	250 Diabetes mellitus	
Hypertension	401–405 Hypertensive disease	C02 (3 + prescriptions/year)
Chronic obstructive pulmonary disease	490–496 COPD and allied conditions	
Cancer (last 3 years)	140–239 Neoplasms	
Dementia	290 Dementia	
	331 Alzheimer’s disease and other cerebral degenerations	
Inflammatory diseases of the central nervous system	320–322 Meningitis	
	323 Encephalitis, myelitis, and encephalomyelitis	
	324 Intracranial and intraspinal abscess	
	325 Phlebitis and thrombophlebitis of intracranial venous sinuses	
	326 Late effects of intracranial abscess or pyogenic infection	
Heart failure	428 Heart failure	
Atrial fibrillation	427.3 Cardiac dysrhythmias	
Inflammatory bowel diseases	555–556	
Rheumatoid diseases	274 Gout	
	696.0 Psoriatic arthropathy	
	710–714 Arthropathies and related disorders	
	720 Spondylarthritis	
	725 Polymyalgia rheumatica	
	729.0 Rheumatism, unspecified; fibrositis; and other disorders of soft tissues	
Liver disease	570 Acute and subacute necrosis of the liver	
	571 Chronic liver disease and cirrhosis	
	572 Liver abscess	
	573 Other disorders of the liver	

**Table A5:** Definitions for ongoing medications.

Medication	Anatomical Therapeutic Chemical (ATC) classification system codes
Corticosteroids	H02
Immunosuppressants	L04
Antibiotics, antivirals, antimycotics	J01, J02, J05, D06AA, and D06AX
Antibiotics	J01, D06AA, and D06AX
Antivirals	J05
Antimycotics	J02
Antiplatelet agents	B01AC*, N02BA01
Anticoagulants (VKAs and DOACs)	B01AA*, B01AE07, B01AF01, B01AF02, B01AF03
NSAIDs	M01A
ACE inhibitors/angiotensin receptor blockers	C09A, C09B, C09C, C09D
Mineralocorticoid receptor antagonists (MRAs)	C03DA
Sacubitril/varsartan	C09DAX
Beta-blockers	C07
Diuretics	C03
Calcium channel blockers	C08C (mainly vascular effects)
	C08D (direct cardiac effects)
Digoxin	C01AA05
Glucose-lowering agents	A10
Sodium-glucose co-transporter 2 (SGLT2) inhibitors	A10BK
Glucagon-like peptide-1 receptor agonists (GLP-1 RA)	A10BJ
Statins	C10AA, C10B
Other lipid-lowering agents	C10AX
Ezetimibe	C10AX09
PCSK9 inhibitors	C10AX13, C10AX14
Omega-3 EPA	C10AX06
Fibrates, resins, nicotinic acid	C10AB, C10AC, and C10AD
Other blood pressure medications	C02 (antihypertensives),
	C08E (non-selective calcium channel blockers),
	C08G (calcium channel blockers and diuretics)
Antigout preparations	M04A
Colchicine	M04AC02

**Table A6:** List of inflammatory diseases (exclusion factor in sensitivity analyses).

Disease	ICD-9 code
Rheumatoid arthritis	714.0
Systemic lupus erythematosus	710.0
Scleroderma	710.1
Sjogren’s syndrome	710.2
Dermatomyositis	710.3
Mixed connective tissue disease	710.9
Behçet's disease	136.1
Polymyalgia rheumatica	725
Ulcerative colitis	556
Crohn's disease	555
Celiac disease	579.0
Psoriasis and psoriatic arthritis	696
Ankylosing spondylitis	720.0
Multiple sclerosis	340
Vasculitis	447.6
