# Supplementary material for: Systemic inflammation prevalence in patients with atherosclerotic cardiovascular disease and chronic kidney disease: a population-based study using a nationwide primary care database in Spain
Source: Front Cardiovasc Med. 2025 Mar 4;12:1538466. doi: 10.3389/fcvm.2025.1538466 (PMC11913838; doi:10.3389/fcvm.2025.1538466)
Supplement: Supplementary file 1 [file Datasheet1.pdf]

Supplementary Tables and Figures

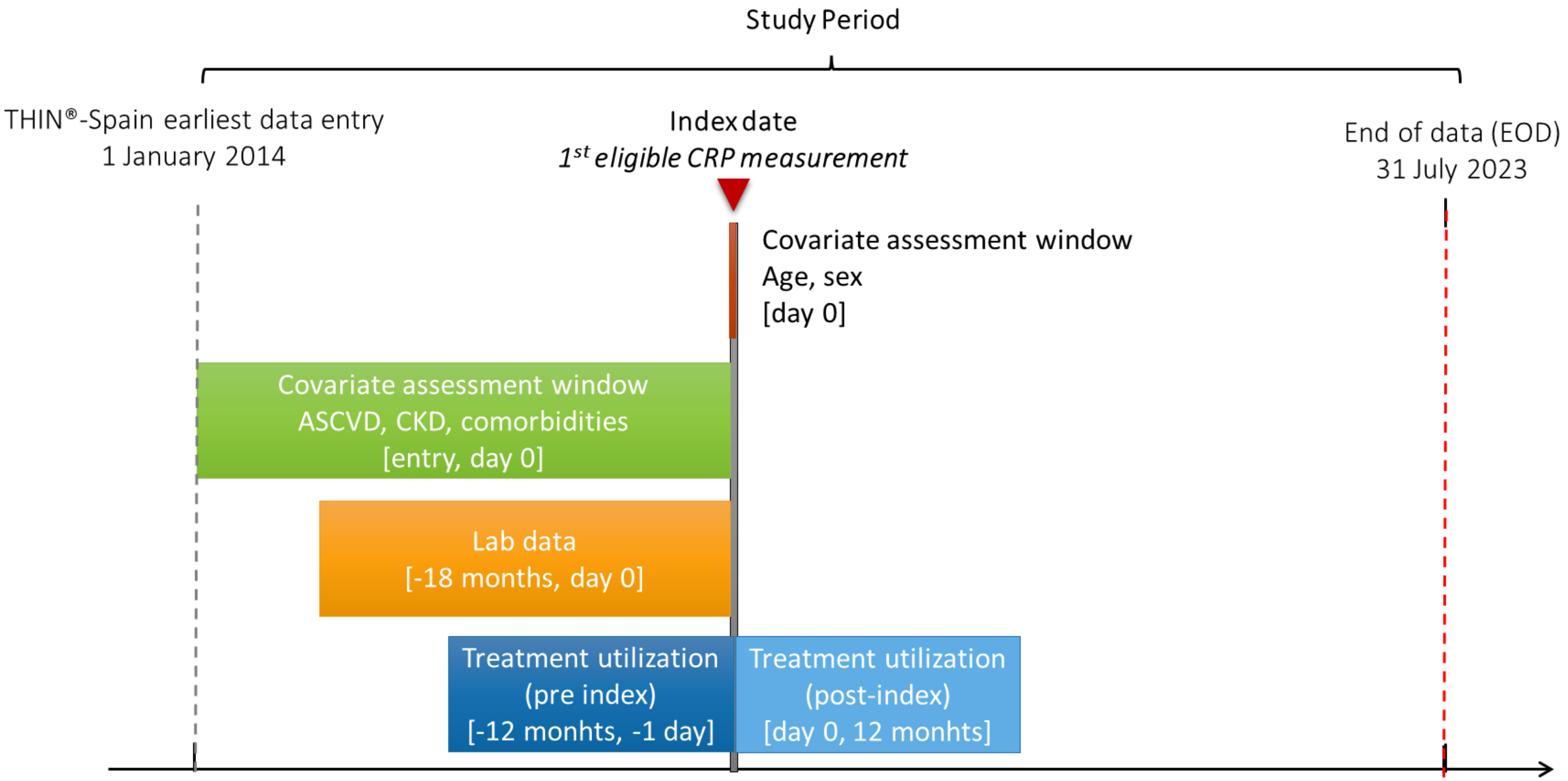

Figure S 1. Diagram of the study design.

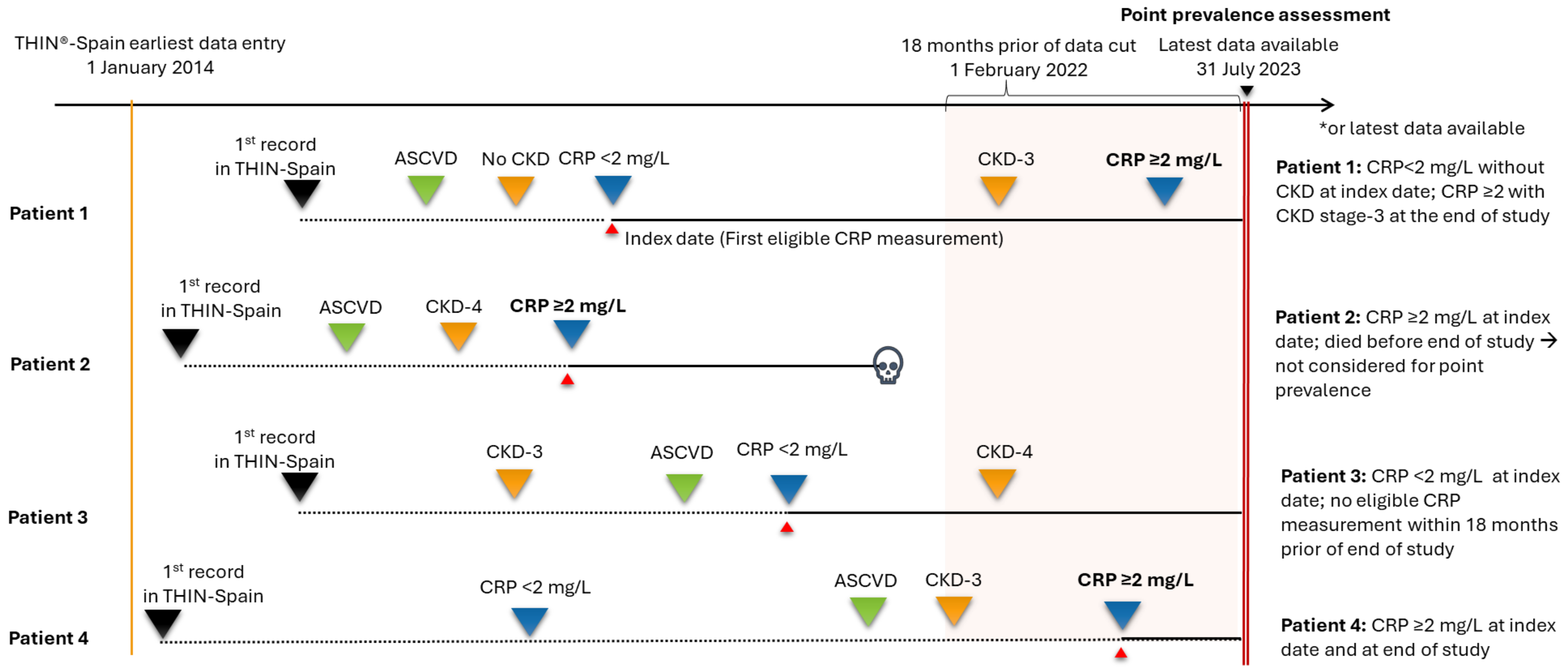

<u>1) Proportion of patients with SI at index date:</u> patients with SI at first eligible CRP measurement/ overall patients included in the study

→ e.g., 2/4 patients = 50%

<u>2) Proportion of patients with SI at the latest available date (point prevalence):</u> patients with SI / "active" patients*

→ e.g., 1/2 patients = 50%

<u>3) Proportion of patients with SI at any point over the study period</u>: patients with SI during study period / overall patients included in the study

→ e.g. 3/4 patients = 75%

*patients alive at the data cut on 31 July 2023 and with ≥1 eligible CRP measurement in the prior 18 months

Figure S 2. **Diagram for the estimation of the proportion of patients with systemic inflammation: 1) at index date (first eligible CRP measurement), 2) at end of study (point prevalence), and 3) over the entire study period.**

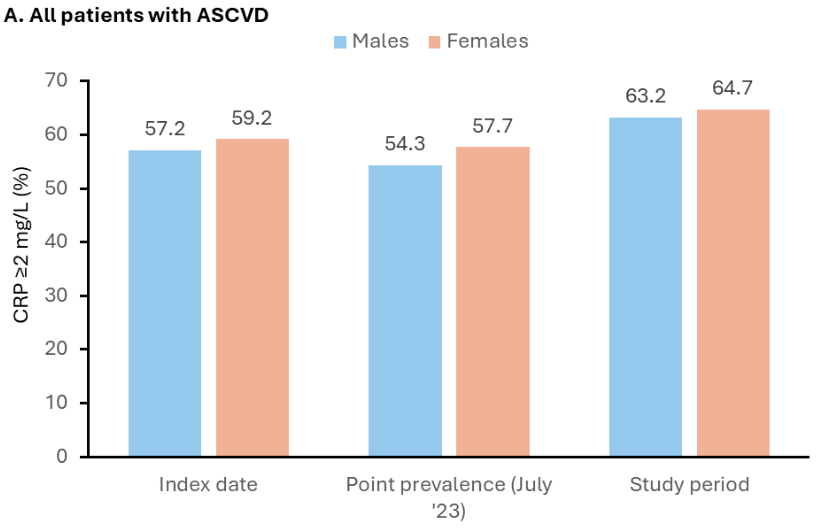

Population denominator (N)	Males	Females
Index date	9,020	6,778
Point prevalence (July 2023)	3,705	2,733
Study period (2014-2023)	9,020	6,778

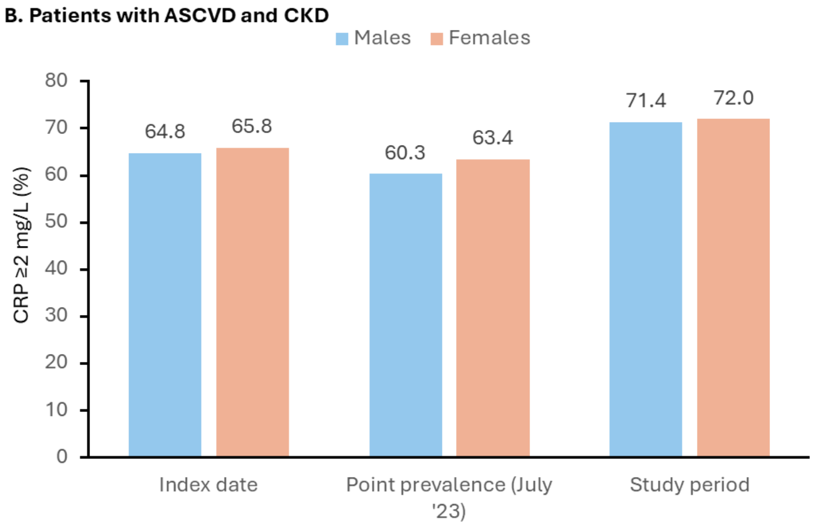

Population denominator (N)	Males	Females
Index date	2,932	2,179
Point prevalence (July 2023)	1,302	955
Study period (2014-2023)	3,348	2,502

Figure S 3. Proportion of systemic inflammation in patients with ASCVD (A) and in patients with ASCVD and comorbid CKD (B), by sex.

A. All patients with ASCVD

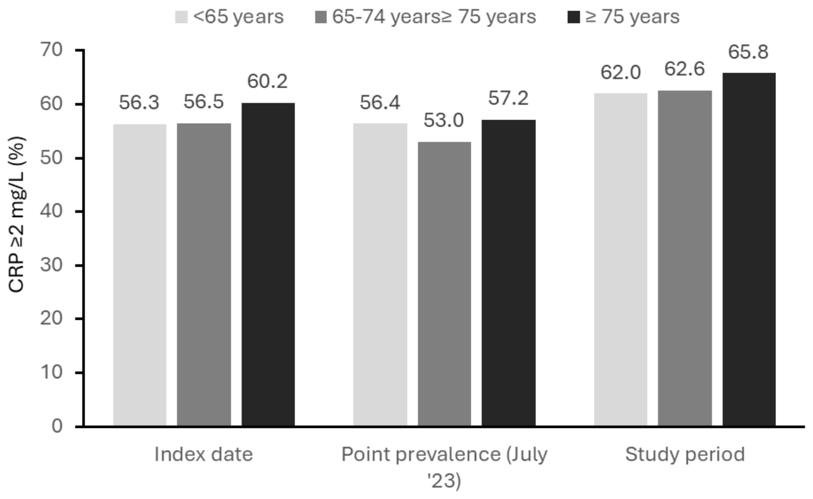

Population denominator (N)	<65 years	65-74 years	≥ 75 years
Index date	4,506	4,496	6,796
Point prevalence (July 2023)	1,974	1,889	4,044
Study period (2014-2023)	4,506	4,496	6,796

B. Patients with ASCVD and CKD

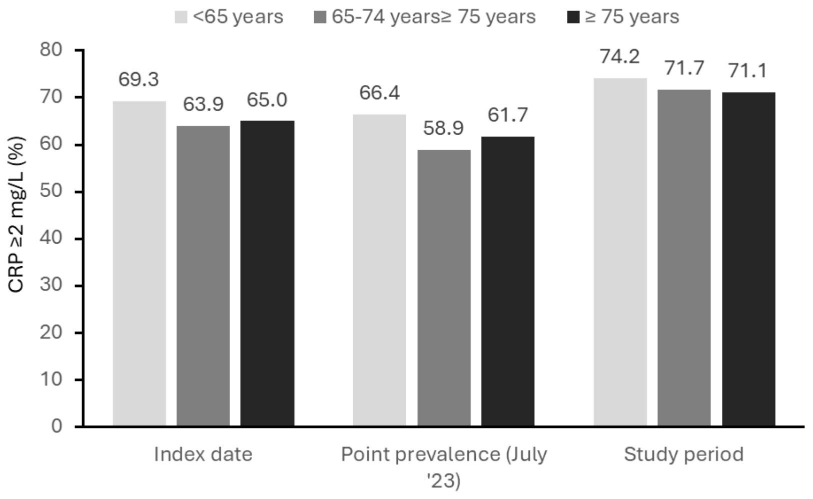

Population denominator (N)	<65 years	65-74 years	≥ 75 years
Index date	547	1,200	3,364
Point prevalence (July 2023)	289	599	1,369
Study period (2014-2023)	685	1,461	3,704

Figure S 4. Proportion of systemic inflammation in patients with ASCVD (A) and in patients with ASCVD and comorbid CKD (B), by age at index date.

Table S 1. Prevalence of systemic inflammation by CKD stage.

	CKD stage 1-2*		CKD stage 3		CKD stage 4		CKD stage 5	
	Eligible patients	Patients with CRP ≥ 2 mg/L	Eligible patients	Patients with CRP ≥ 2 mg/L	Eligible patients	Patients with CRP ≥ 2 mg/L	Eligible patients	Patients with CRP ≥ 2 mg/L
	N	%	N	%	N	%	N	%
**Proportion of patients with systemic inflammation at first eligible CRP measurement**	905	64.1%	3568	64.6%	493	70.8%	54	72.2%
**Point prevalence at study data cut (July 31, 2023)**	434	61.1%	1554	60.9%	241	66.4%	<30	72.2%
- Deaths before study data cut	161		863		213		35	
- No eligible CRP measurements within 18 months from data cut	471		1599		177		<30	
**Proportion of patients with systemic inflammation at any time during the study period (2014-2023)**	1066	69.7%	4016	71.1%	631	78.0%	71	77.5%

*CKD stage 1-2: CKD patients with eGFR >60 mL/min/1.73m^2 but UACR > 30 mg/g. CKD progression during the study period has been considered, i.e., the proportion of patients with CKD during the entire study period is higher than the proportion of patients with CKD at first eligible CRP measurement.

Table S 2. Drug utilisation in patients with ASCVD, by systemic inflammation (SI) status. The table reports the % of patients using each pharmacological group in the 12 months after the first eligible CRP measurement (index date).

	All ASCVD	ASCVD with SI	ASCVD without SI	
	N= 15,798	N=9,169	N=6,629	
	%	%	%	p-value*
Corticosteroids	8.1%	9.2%	6.5%	**<.001**
Immunosuppressants	0.6%	0.8%	0.3%	**<.001**
Antibiotics, antivirals, antimycotics	40.2%	42.9%	36.4%	**<.001**
- Antibiotics	39.2%	41.9%	35.4%	**<.001**
- Antivirals	1.2%	1.1%	1.3%	0.175
- Antimycotics	1.2%	1.4%	1.0%	**0.010**
Antiplatelet agents	64.7%	63.9%	65.9%	**0.009**
- *Aspirin*	54.3%	53.2%	55.6%	**0.003**
Anticoagulants (VKAs and DOACs)	14.9%	16.7%	12.5%	**<.001**
NSAIDs	27.7%	28.2%	26.9%	0.062
ACEi/ARBs	61.6%	63.7%	58.8%	**<.001**
MRAs	5.8%	6.6%	4.7%	**<.001**
Beta-blockers	44.6%	45.3%	43.7%	0.054
Diuretics	26.7%	30.6%	21.3%	**<.001**
Calcium Channel Blockers	20.5%	21.5%	19.1%	**<.001**
Glucose lowering agents	32.4%	34.1%	30.0%	**<.001**
- SGLT2 inhibitors	5.8%	6.2%	5.2%	**0.005**
- GLP-1 receptor agonists	2.0%	2.3%	1.6%	**0.001**
Statins	72.2%	69.6%	75.9%	**<.001**
Other lipid lowering agents	3.7%	3.4%	4.1%	**0.020**
- Ezetimibe	3.3%	3.0%	3.7%	**0.013**
- PCSK9 inhibitors	0.4%	0.3%	0.5%	**0.021**
- Omega 3	0.2%	0.3%	0.1%	0.087
Fibrates, Resins, Nicotinic acid	5.1%	5.5%	4.5%	**0.007**
Other blood pressure medications	4.0%	4.4%	3.4%	**0.001**
Colchicine	1.7%	2.0%	1.3%	**0.001**

ASCVD: atherosclerotic cardiovascular disease; CKD: chronic kidney disease; SI: systemic inflammation; VKA: Vitamin K antagonists; DOAC: direct oral anticoagulant; NSAID: non-steroid anti-inflammatory drugs; ACEi: angiotensin-converting enzyme inhibitor; ARB: angiotensin II receptor blocker; MRA: mineralocorticoid receptor antagonist; SGLT2: Sodium-glucose cotransporter-2; GLP-1: Glucagon-like peptide-1; PCSK9: Proprotein Convertase Subtilisin/Kexin type-9.

Table S 3. Drug utilisation in patients with ASCVD and CKD, by systemic inflammation (SI) status. The table reports the % of patients using each pharmacological group in the 12 months after the first eligible CRP measurement.

	**All ASCVD+CKD**	**ASCVD+CKD, With SI**	**ASCVD+CKD, Without SI**	
	N=5,111	N=3,334	N=1,777	
	%	%	%	p-value*
Corticosteroids	8.5%	9.7%	6.4%	**<.001**
Immunosuppressants	0.5%	0.6%	0.3%	0.120
Antibiotics, antivirals, antimycotics	44.7%	47.3%	39.8%	**<.001**
- Antibiotics	43.9%	46.3%	39.3%	**<.001**
- Antivirals	1.0%	1.0%	1.0%	0.981
- Antimycotics	1.2%	1.3%	0.8%	0.130
Antiplatelet agents	64.8%	63.9%	66.6%	0.058
- *Aspirin*	52.6%	51.9%	53.8%	0.193
Anticoagulants (VKAs and DOACs)	22.8%	24.2%	20.1%	**0.001**
NSAIDs	20.8%	21.3%	20.0%	0.302
ACEi/ARBs	72.8%	72.5%	73.2%	0.599
MRAs	9.5%	10.5%	7.7%	**0.001**
Beta-blockers	52.8%	52.8%	52.6%	0.874
Diuretics	43.5%	47.0%	36.9%	**0.000**
Calcium Channel Blockers	27.6%	28.2%	26.4%	0.191
Glucose lowering agents	46.4%	46.5%	46.1%	0.784
- SGLT2 inhibitors	9.1%	9.4%	8.6%	0.340
- GLP-1 receptor agonsists	3.0%	3.2%	2.6%	0.215
Statins	74.2%	71.5%	79.2%	**<.001**
Other lipid lowering agents	3.6%	3.7%	3.6%	0.916
- Ezetimibe	3.2%	3.1%	3.3%	0.824
- PCSK9 inhibitors	0.3%	0.2%	0.4%	0.333
- Omega 3	0.4%	0.4%	0.2%	0.263
Fibrates, Resins, Nicotinic acid	7.0%	7.3%	6.5%	0.259
Other blood pressure medications	7.4%	7.6%	7.0%	0.427
Colchicine	2.3%	2.6%	1.5%	**0.010**

ASCVD: atherosclerotic cardiovascular disease; CKD: chronic kidney disease; SI: systemic inflammation; VKA: Vitamin K antagonists; DOAC: direct oral anticoagulant; NSAID: non-steroid anti-inflammatory drugs; ACEi: angiotensin-converting enzyme inhibitor; ARB: angiotensin II receptor blocker; MRA: mineralocorticoid receptor antagonist; SGLT2: Sodium-glucose cotransporter-2; GLP-1: Glucagon-like peptide-1; PCSK9: Proprotein Convertase Subtilisin/Kexin type-9.

Table S 4. Prevalence of systemic inflammation in patients with ASCVD and without chronic inflammatory diseases, by CKD status.

	**Overall**		**Patients without CKD**		**Patients with CKD**	
	Eligible patients	Patients with CRP ≥ 2 mg/L	Eligible patients	Patients with CRP ≥ 2 mg/L	Eligible patients	Patients with CRP ≥ 2 mg/L
	N	%	N	%	N	%
**Patients with systemic inflammation at first eligible CRP measurement**	14546	57.6%	8944	54.0%	4695	65.0%
**Point prevalence at data cut (July 31, 2023)**	5913	55.5%	3712	51.8%	2077	61.4%
(Patients deceased before data cut)	2058		761		1217	
(Patients without CRP measurement in the latest 18 months)	6575		4094		2099	
**Patients with systemic inflammation at any time during the study period (Jan 2014- Jul 2023)***	14546	63.5%	8567	58.8%	5393	71.5%

CKD progression during the study period has been considered, i.e., the proportion of patients with CKD during the entire study period is higher than the proportion of patients with CKD at first eligible CRP measurement.

Table S 5. Characteristics of patients with ASCVD, with and without CRP measurement during the study period. Data expressed as mean (standard deviation) or percentage.

**Variable**	**Patients with CRP**	**Patients without CRP**	p-value
	N=26,082	N=48,775	
Age (years)	69.1 (12.3)	69.9 (13.7)	<.001
Female sex	44.23%	38.11%	<.001
Current smoker	22.21%	19.05%	<.001
BMI (kg/m2)	29.58 (5.41)	29.47 (5.39)	0.158
**ASCVD type**			<.001
Coronary disease	49.51%	51.97%	<.001
Cerebrovascular disease	41.16%	40.99%	0.650
PAD	4.89%	4.25%	<.001
Other	8.37%	7.40%	<.001
**Comorbidities**			<.001
CKD	16.08%	9.23%	<.001
Diabetes	28.89%	26.03%	<.001
Hypertension	55.62%	48.39%	<.001
COPD	21.42%	17.48%	<.001
Cancer*	1.57%	1.20%	<.001
Dementia	3.26%	4.96%	<.001
Heart failure	6.18%	7.19%	<.001
Atrial fibrillation	8.57%	8.30%	0.210
IBD	0.67%	0.40%	<.001
Rheumatoid disease	13.96%	10.02%	<.001
CNS inflammatory disease	0.10%	0.09%	0.510
Liver disease	3.20%	1.75%	<.001

ASCVD: atherosclerotic cardiovascular disease; CRP: C-reactive protein; BMI: body mass index; PAD: peripheral artery disease; COPD: chronic obstructive pulmonary disease; IBD: inflammatory bowel disease; CNS: central nervous system.
